# LncRNA OIP5-AS1 promotes the malignancy of pancreatic ductal adenocarcinoma via regulating miR-429/FOXD1/ERK pathway

**DOI:** 10.1186/s12935-020-01366-w

**Published:** 2020-07-09

**Authors:** Liping Wu, Yongcun Liu, Cheng Guo, Yuan Shao

**Affiliations:** 1 Department of Endocrinology, The First Affiliated Hospital of Xi’an Jiaotong University, No. 277 West Yanta Road, Xi’an, 710061 Shaanxi China; 2 Department of Oncology, The First People’s Hospital of Xianyang, Xianyang, 712000 Shaanxi China; 3 Department of Hepatobiliary Surgery, The First Affiliated Hospital of Xi’an Jiaotong University, Xi’an, 710061 Shaanxi China; 4 Department of E.N.T, The First Affiliated Hospital of Xi’an Jiaotong University, Xi’an, 710061 Shaanxi China

**Keywords:** OIP5-AS1, miR-429, FOXD1, Proliferation, ERK pathway, PDAC

## Abstract

**Background:**

Pancreatic ductal adenocarcinoma (PDAC), a subtype of pancreatic cancer, is a malignant tumor with unfavorable prognosis. Despite accumulating researches have made efforts on finding novel therapeutic methods for this disease, the underlying mechanism of long non-coding RNAs (lncRNAs) remains elusive. OIP5 antisense RNA 1 (OIP5-AS1) has been reported to play important role in the occurrence and development of multiple human cancers. This study was aimed at unveiling the regulatory role of OIP5-AS1 in PDAC.

**Methods:**

RT-qPCR analysis revealed the OIP5-AS1 expression in PDAC tissues and adjacent normal ones. Kaplan–Meier method was applied to analyze the overall survival of patients with high or low level of OIP5-AS1. Gain- or loss-of function assays were performed to assess the effects of OIP5-AS1 knockdown on cell functions, including proliferation, migration and EMT process. Mechanism experiments, such as luciferase reporter and RNA pull-down assays proved the interaction between OIP5-AS1 and miR-429 as well as that between miR-429 and FOXD1.

**Results:**

OIP5-AS1 was up-regulated in PDAC tissues and cell lines, and high level of OIP5-AS1 indicated poor prognosis in PDAC patients. OIP5-AS1 knockdown hindered cell proliferation, migration and epithelial-mesenchymal transition (EMT) process, while overexpression of OIP5-AS1 caused the opposite results. OIP5-AS1 activated ERK pathway through up-regulating forkhead box D1 (FOXD1) expression by sponging miR-429. Furthermore, OIP5-AS1 facilitated cell growth in vivo.

**Conclusion:**

OIP5-AS1 exerted oncogenic function in PDAC cells through targeting miR-429/FOXD1/ERK pathway.

## Background

Pancreatic cancer (PC) is identified as one of the most aggressively malignant cancers and the fourth main reason for cancer-associated death worldwide [1, 2]. Although great improvement has been made in therapeutic methods, the survival rate of PC patients remains poor [3, 4]. Pancreatic ductal adenocarcinoma (PDAC) is the main subtype of pancreatic cancer. Despite mounting researches on PDAC, the molecular mechanisms associated with the tumorigenesis and progression remain to be explored. Hence, exploring the potential molecular mechanisms of PDAC progression is of great necessity for finding effective biomarkers for PDAC treatment.

Long non-coding RNAs (lncRNAs), possessing over 200 nucleotides, are a type of RNA molecules without protein-coding capacity [5]. Previous researches have elucidated that lncRNAs exert critical roles in various biological processes, such as cell growth, cell apoptosis and metastasis [6, 7]. Extensive literatures indicated that lncRNAs function as tumor promoters or inhibitors in PDAC. For example, lncRNA PVT1 triggers autophagy and progress in PDAC [8]. LncRNA UCA1 could enhance the proliferative and anti-apoptotic abilities of PDAC cells [9]. Exosomal lncRNA Sox2ot promotes stemness in PDAC [10]. Knockdown of lncRNA MEG3 increases cell proliferation, migration, invasion, sphere-formation ability and cancer stem cell properties in PDAC [11]. Recently, lncRNA OIP5-AS1 has been demonstrated to be dysregulated and promote tumorigenesis in diverse cancer types, including cervical cancer, lung cancer, hepatoblastoma and colorectal cancer [12–15]. Nevertheless, the potential role of OIP5-AS1 in PDAC is still unclear.

According to a large number of studies, lncRNAs could play the role of competing endogenous RNAs (ceRNAs) or molecular sponges for microRNAs (miRNAs) to regulate gene expression [16]. MiRNAs are a cluster of little non-coding RNAs with around 18–25 nucleotides, acting as post-transcriptional regulators [17, 18]. More and more evidences have demonstrated the crucial roles of miRNAs in human cancers [19–21]. MiR-429 is a cancer-related miRNA whose aberrant expression has been uncovered in many cancers. For example, miR-429 reduces cell migration and invasion in breast cancer [22]. MiR-429 modulates tumor progression in colorectal cancer [23]. More importantly, miR-429 has been revealed to improve prognosis and inhibit tumorigenesis in PDAC via targeting TBK1 [24]. Nevertheless, the modulatory relationship between OIP5-AS1 and miR-429 in PDAC remains veiled.

Current work was aimed to unveil the role of OIP5-AS1 in PDAC and investigated the underlying molecular mechanisms.

## Methods

### Clinical samples

Total of 110 pairs of PDAC tissues (cancerous and matched non-tumor ones) were collected from PDAC patients who underwent surgery at the First Affiliated Hospital of Xi’an Jiaotong University and then stored at − 80° after immediate frozen via liquid nitrogen. All patients signed the written informed consents for this study. The approvals for this study were received from the Clinical Research Ethics Committee of the First Affiliated Hospital of Xi’an Jiaotong University.

### Cell culture and plasmids transfection

Human PDAC cell lines (PANC-1, BxPC-3, AsPC-1 and CFPAC-1) and normal human pancreatic cells (HPDE6-C7) were provided by the American Type Culture Collection (ATCC, Manassas, VA, USA). Above cells were incubated in Dulbecco’s Modified Eagle Medium (DMEM; Gibco, Carlsbad, CA, USA) supplemented with 10% fetal bovine serum (FBS) in humidified air at 37 °C with 5% CO_2_.

Short hairpin RNAs (shRNAs) for OIP5-AS1 knockdown (sh-OIP5-AS1-1 and sh-OIP5-AS1-2) and negative control (sh-NC), the pcDNA3.1 vector for the overexpression of OIP5-AS1 and FOXD1 (pcDNA3.1/OIP5-AS1 and pcDNA3.1/FOXD1) and corresponding control (pcDNA3.1 vector) were constructed by GenePharma. MiR-429 mimic, inhibitor and their negative control (miR-NC) were also prepared by GenePharma. Thereafter, the transfection of all above plasmids was conducted by utilizing Lipofectamine 3000 (Invitrogen, Carlsbad, CA, USA) under the manufacturer’s instruction.

### RNA extraction and quantitative real-time polymerase chain reaction (RT-qPCR)

Total RNA was extracted from PDAC tissues or cells by use of TRIzol Reagent (Invitrogen). Afterwards, RNAs were reversely transcribed into cDNA with TaqMan™ Advanced miRNA cDNA Synthesis Kit (for mRNA and lncRNA; Waltham, MA, USA) or First Strand cDNA Synthesis Kit (for miRNA; Takara, Otsu, Japan). The RT-qPCR analysis was conducted on ABI 7500 Fast Real-Time polymerase chain reaction system (Applied Biosystems, Foster City, CA, USA) via using SYBR Green PCR Kit (Takara, Japan). The relative expression of genes was calculated with the 2^−ΔΔCt^ method. All data were normalized to the expression of U6 or GAPDH.

### Cell Counting Kit-8 (CCK-8) assay

To detect cell proliferation, CCK-8 Kit (Beyotime, Shanghai, China) was used in this experiment under manufacturer’s protocols. Cells (1000 cells/well) were seeded and incubated in 96-well plates. After 24, 48, 72 and 96 h, the CCK-8 reagents were supplemented to each well. Detection of cell viability was performed with a microplate reader at the wave length of 450 nm.

### Colony formation assay

Cells (1000 cells/well) were placed into six-well plates, and then maintained in DMEM medium with 10% FBS. Following incubation for 2 weeks, 10% formaldehyde was used to fix the cells, and then 0.1% crystal violet (Sigma, USA) was applied to stain cells. Finally, the number of visible colonies (with over 50 cells) was calculated manually.

### Transwell assay

For migration assay, cells were incubated in non-serum medium (200 μL) and placed in the upper transwell chambers (Corning Costar, NY, USA). 600 μL DMEM medium containing 20% FBS was supplemented into the bottom chamber. Post 24 h of incubation, cells remained in the upper chamber were removed, while cells migrated to the lower chamber were fixated by methanol and stained via crystal violet. Finally, cells in five randomly assigned fields were calculated under microscope.

### RNA immunoprecipitation (RIP) assay

RIP experiments were performed via utilizing EZ-Magna RIP™ RNA Binding Protein Immunoprecipitation Kit (17-701, Millipore). Considering the importance of Ago2 in RNA-induced silencing complexes (RISCs) [25], we applied Ago2 antibodies to conduct this experiment. In brief, cells were isolated and lysed with RIP buffer supplemented with RNase inhibitor. RIP buffer containing magnetic beads conjugated with Ago2 antibody was used to incubate 100 μl of whole cell extract. The precipitated RNAs were eluted and then measured by RT-qPCR. Input containing 10% cell lysates acted as the positive control and antibodies targeting IgG were the negative control. Besides, RNAs precipitated in these three groups were also subjected to agarose gel electrophoresis after semi-quantitative PCR.

### RNA pull down assay

RNA pull-down assay was performed using the Pierce Magnetic RNA–Protein Pull-Down Kit (Thermo Fisher Scientific, Waltham, MA, USA). miR-429 sequence was labeled with biotin probe. Non-biotin labeled sequence was used as the negative control. Subsequently, cell lysates were incubated with biotin-miR-429 probe or non-biotin probe and then incubated with the magnetic beads for 30 min. The complex was washed and RNAs were purified with TRIzol reagent (Thermo Fisher Scientific). RNA enrichment was measured by RT-qPCR.

### Luciferase reporter assay

The sequences of wide-type OIP5-AS1 or mutant OIP5-AS1 with or without the conjectured miR-429 binding sites were cloned and inserted into pmirGLO dual-luciferase reporters (Promega, Madison, WI, USA) to construct the pmirGLO-OIP5-AS1-wild type (OIP5-AS1-Wt) or pmirGLO-OIP5-AS1-mutant (OIP5-AS1-Mut) reporter vector. Similarly, sequences of wild-type or mutant-type 3′UTR of FOXD1 mRNA with or without the predicted miR-429 binding sites were cloned and inserted into pmirGLO vector (Promega) to construct the pmirGLO-FOXD1-wild type (FOXD1-Wt) or pmirGLO-FOXD1-mutant (FOXD1-Mut) reporter vector. Thereafter, above recombinant plasmids were co-transfected into PANC-1 and CFPAC-1 cells with miR-NC, miR-429 mimic or miR-429 mimic + pcDNA3.1/OIP5-AS1 by the use of Lipofectamine 3000, as needed. After transfection for 48 h, the Dual-Luciferase Reporter Assay System (Promega) was applied to detect the luciferase activity. Transfected cells were lysed and added with the luciferin that was catalyzed with luciferase. The luciferase intensity was examined at the wavelength of 560 nm. The luciferase activity of each reporter vector was normalized to Renilla luciferase activity (465 nm), and then the luciferase activity of each reporter in NC mimics was set as 1.0, that in miR-429 mimics group or miR-429 mimic + pcDNA3.1/OIP5-AS1 group was relative to NC mimics group.

### Western blot

Cells were lysed by Radio Immunoprecipitation Assay (RIPA) buffer (Radio-Immunoprecipitation assay buffer, Beyotime). Then, bicinchoninic acid (BCA) Protein Assay Kit (Beyotime) was applied to detect the concentrations of above protein samples. Afterwards, proteins were segregated with the 10% sodium dodecyl sulfate–polyacrylamide gel electrophoresis (SDS-PAGE) and then transferred onto polyvinylidene difluoride (PVDF) membranes (Millipore, Boston, MA, USA). After blocking, membranes were incubated with primary antibodies against FOXD1 (ab49156; 1:1000 dilution), E-cadherin (ab40772; 1:10,000 dilution), N-cadherin (ab18203; 1:1000 dilution), Vimentin (ab45939; 1:1000 dilution), ERK1/2 (ab17942; 1:1000 dilution), phosphorylated ERK1/2 (ab223500; 1:1000 dilution) and GADPH (ab8245; 1:10,000 dilution) at 4 °C overnight. All antibodies were provided by Abcam Company (Cambridge, UK). Subsequently, the membranes were cultured with the corresponding secondary antibodies, followed by analysis of the bands by the enhanced chemiluminescence (ECL) detection system (Pierce Biotechnology, Rockford, IL, USA).

### In vivo experiment

Before the in vivo experiment, stably transfected PDAC cells with sh-OIP5-AS1-1 or pcDNA3.1/OIP5-AS1 were established. Parental cells were seeded in 96 well plates at 37 °C for 12 h. Next, puromycin at different final concentrations (2, 4, 6, 8, 10 μg/ml) was added into the wells. The reagent was changed every 2–3 days. Seven days later, the lowest concentration led to the sacrifice of cells was determined. Afterwards, cells were infected with the lentivirus containing sh-OIP5-AS1, sh-NC, pcDNA3.1 or pcDNA3.1/OIP5-AS1. Ten days later, stably transfected cells were selected out for animal study.

To carry out the vivo tumorigenesis assay, 20 BALB/c nude mice (4-week-old, 15–20 g) were procured from National Laboratory Animal Center (Beijing, China). The animal experimental procedures were carried out strictly conforming to the protocol approved by the Administrative Panel on Laboratory Animal Care of the First Affiliated Hospital of Xi’an Jiaotong University. PANC-1 cells (5 × 10^6^) transfected with sh-OIP5-AS1-1 or sh-NC as well as CFPAC-1 cells (1 × 10^6^) with or without OIP5-AS1 overexpression in PBS media were subcutaneously injected into the mice. N = 5 in each group. The volume of tumors was monitored using a Vernier caliper every 4 days. Four weeks later, mice were euthanatized. The weight of tumors was recorded. Tumor volume = 1/2 × length × width^2^.

### Immunohistochemistry (IHC) staining

The paraffin-embedded tumor tissues were dewaxed and subsequently incubated with the primary antibodies against FOXD1 (Cell Signaling Technology, USA) and Ki-67 (Cell Signaling Technology, USA) at 4 °C overnight, followed by incubation of secondary antibodies in accordance with the manufacturer’s protocols. To visualize the staining of indicated proteins, samples were incubated with 3,3′-diaminobenzidine in 0.1% H_2_O_2_ in Tris–HCl buffer and then counterstained with Hematoxylin QS (Vector Laboratories).

### Statistical analysis

Data was presented as the mean ± standard deviation (S.D.). Each experiment was carried out for 3 times. SPSS 22.0 software (SPSS, Chicago, IL, USA) was used to analyze all the results collected. The overall survival curve was generated using Kaplan–Meier method and the log-rank test. Student’s t test or one-way/two-way analysis of variance (ANOVA) with post hoc test (Turkey’s test) was used to evaluate differences between groups. Pearson’s correlation analysis was applied to determine the expression correlations. P < 0.05 was thought as statistically significant.

## Results

### OIP5-AS1 is up-regulated in PDAC tissues and predicts poor prognosis of PDAC patients

**Fig. 1:**
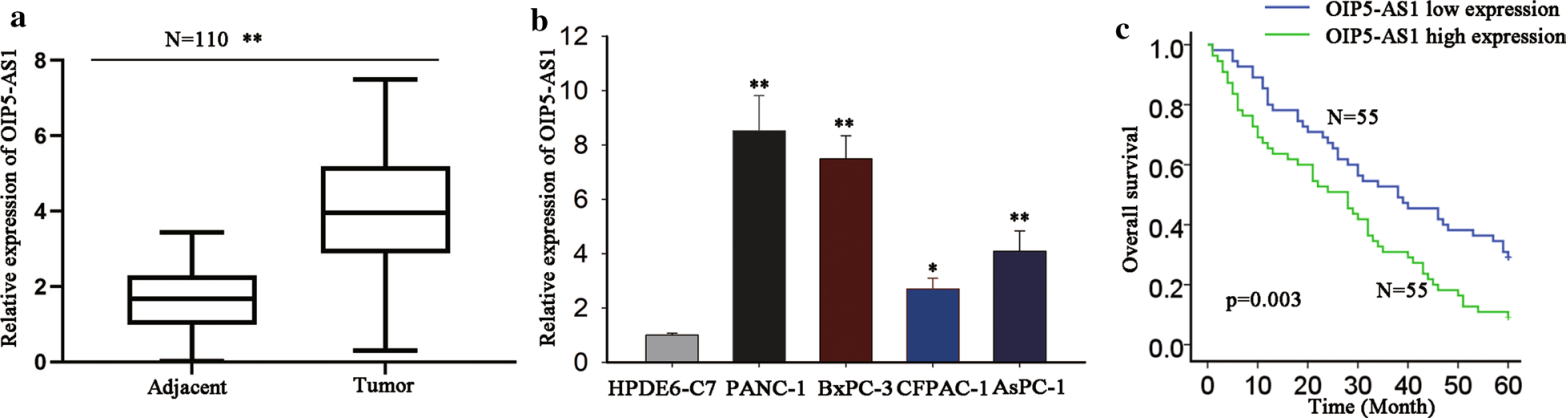
OIP5-AS1 is upregulated in PDAC tissues and cells and predicts prognosis of PDAC patients. **a** OIP5-AS1 expression was analyzed in 110 PDAC tissues by RT-qPCR (**P < 0.01). **b** The relative expression of OIP5-AS1 in PDAC cells (PANC-1, BxPC-3, AsPC-1 and CFPAC-1) was significantly upregulated in comparison to normal pancreatic cells (HPDE6-C7). (*P< 0.05, and **P < 0.01.) **c** Kaplan–Meier analysis validated that higher OIP5-AS1 expression resulted in more unsatisfactory overall survival of PDAC patients (P = 0.003). *P< 0.05, and **P < 0.01 indicated data were statistically significant

At first, high level of OIP5-AS1 in TCGA PAAD samples was obtained from GEPIA (Additional file 1: Fig. S1A). Importantly, high level of OIP5-AS1 had close correlation with poor prognosis in PAAD patients (Additional file 1: Fig. S1B). Moreover, OIP5-AS1 expression in 110 pairs of tissues (adjacent normal and tumor) was detected by RT-qPCR. As a result, we observed remarkable the up-regulation of OIP5-AS1 in PDAC tissues compared to adjacent normal ones (Fig. 1a). Additionally, enhanced level of OIP5-AS1 was also unveiled in four PDAC cell lines (PANC-1, BxPC-3, AsPC-1 and CFPAC-1) in comparison with normal HPDE6-C7 cells (Fig. 1b). According to the median value of OIP5-AS1 level in 110 samples, all patients were divided into two groups (OIP5-AS1 high or low expression group). Additionally, the correlation between OIP5-AS1 level and clinicpathological characteristics of PDAC patients was evaluated. We found that high OIP5-AS1 level was positively associated with tumor size, distant metastasis and TNM stage (Table 1). Moreover, Kaplan–Meier survival analysis [26] illustrated that PDAC patients with high level of OIP5-AS1 exhibited poorer overall survival (Fig. 1c). Besides, OIP5-AS1 expression and TNM stage were validated as the independent prognostic markers for PDAC patients (Table 2). Collectively, OIP5-AS1 might function as an oncogene in PDAC.

**Table 1:** Correlation between OIP5-AS1 expression and the clinical characteristic of patients with pancreatic cancer. (n = 110)

Variable	OIP5-AS1 expression		P-value
	Low	High	
Age			
< 60	36	33	0.694
≥ 60	19	22	
Gender			
Male	36	38	0.839
Female	19	17	
Tumor size (cm)			
< 3	36	15	< 0.001***
≥ 3	19	40	
Differentiation			
Well/moderate	25	23	0.848
Poor	30	32	
Distant metastasis			
Absent	35	15	< 0.001***
Present	20	40	
TNM stage			
I/II	31	14	0.002**
III/IV	24	41	

Low/high by the sample median. Pearson χ^2^ test

**P < 0.01, ***P < 0.001 was considered statistically significant

**Table 2:** Multivariate analysis of prognostic parameters in patients with pancreatic cancer by Cox regression analysis

Variable	Category	P-value
Age		
	< 60	0.201
	≥ 60	
Gender		
	Male	0.205
	Female	
Tumor size (cm)		
	< 3	0.523
	≥ 3	
Differentiation		
	Well/moderate	0.191
	Poor	
Distant metastasis		
	Absent	0.478
	Present	
TNM stage		
	I–II	0.005**
	III–IV	
OIP5-AS1 level		
	Low	0.013*
	High	

Proportional hazards method analysis showed a positive, independent prognostic importance of OIP5-AS1 expression (P = 0.013). *P < 0.05, **P < 0.01 was considered statistically significant

### Silencing of OIP5-AS1 suppresses PDAC cell growth, migration and reversed EMT process

**Fig. 2:**
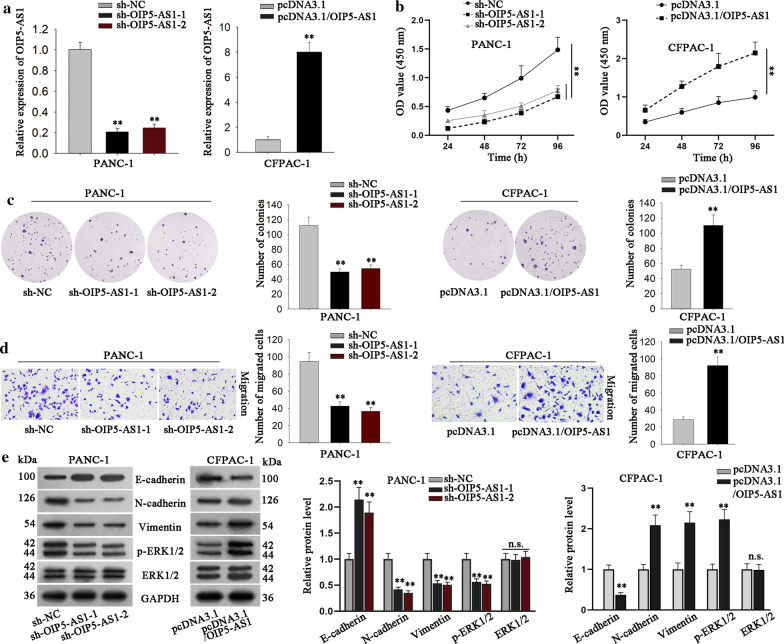
OIP5-AS1 promotes PDAC cell proliferation and migration. **a** The level of OIP5-AS1 was measured in PANC-1 cells transfected with sh-OIP5-AS1-1, sh-OIP5-AS1-2 or CFPAC-1 cells transfected with pcDNA3.1 empty vector or pcDNA3.1/OIP5-AS1. Results were evaluated by RT-qPCR (**P < 0.01). The transfected cells were used for subsequent experiments. **b**, **c** CCK-8 and colony formation assays detected measured the cell proliferation ability in indicated groups. (**P < 0.01). **d** Transwell assay detected cell migration ability in PANC-1 cells transfected with indicated plasmids (**P < 0.01). **e** Western blot researched the effects of OIP5-AS1 depletion or up-regulation on proteins involved in EMT process and ERK signaling pathway in cells under different conditions (**P < 0.01). *P< 0.05, and **P < 0.01 indicated data were statistically significant

Loss- and gain-of-function assays were then implemented to investigate the precise function of OIP5-AS1 in PDAC. Prior to that, we confirmed the silencing of OIP5-AS1 by shRNAs (sh-OIP5-AS1-1/2) in PANC-1 cells and the overexpression of OIP5-AS1 by pcDNA3.1/OIP5-AS1 in CFPAC-1 cells (Fig. 2a). Subsequently, CCK-8 assay elucidated that OIP5-AS1 silence conspicuously inhibited the proliferative ability of PANC-1 cells, while OIP5-AS1 overexpression led to contrary results, and colony formation assay further proved above results (Fig. 2b, c). Also, Transwell assay detected that OIP5-AS1 inhibition obviously suppressed the migratory ability of PANC-1 cells, whereas OIP5-AS1 overexpression led to the opposite changes in CFPAC-1 cells (Fig. 2d). Furthermore, the enhanced E-cadherin level and decreased levels of N-cadherin and Vimentin were detected in OIP5-AS1-silenced PANC-1 cells whereas the opposite results were observed in OIP5-AS1-overexpressed CFPAC-1 cells (Fig. 2e, lane 1–3). Recently, researchers have revealed that extracellular signal-regulated kinase (ERK) pathway has important effects on the initiation and maintenance of PDAC [27–29]. On this basis, we wondered whether OIP5-AS1 regulated PDAC progression through regulating this pathway. As presented in Fig. 2e (lane 4–5), the activity of ERK1/2, the key proteins of ERK signaling pathway, was positively regulated by OIP5-AS1 since the phosphorylation of ERK1/2 was hindered by knockdown of OIP5-AS1 but strengthened under OIP5-AS1 overexpression. These evidences demonstrated that OIP5-AS1 promoted cell proliferation, migration and EMT process and activated ERK pathway in PDAC.

### MiR-429 is negatively regulated by OIP5-AS1 in PDAC cells

**Fig. 3:**
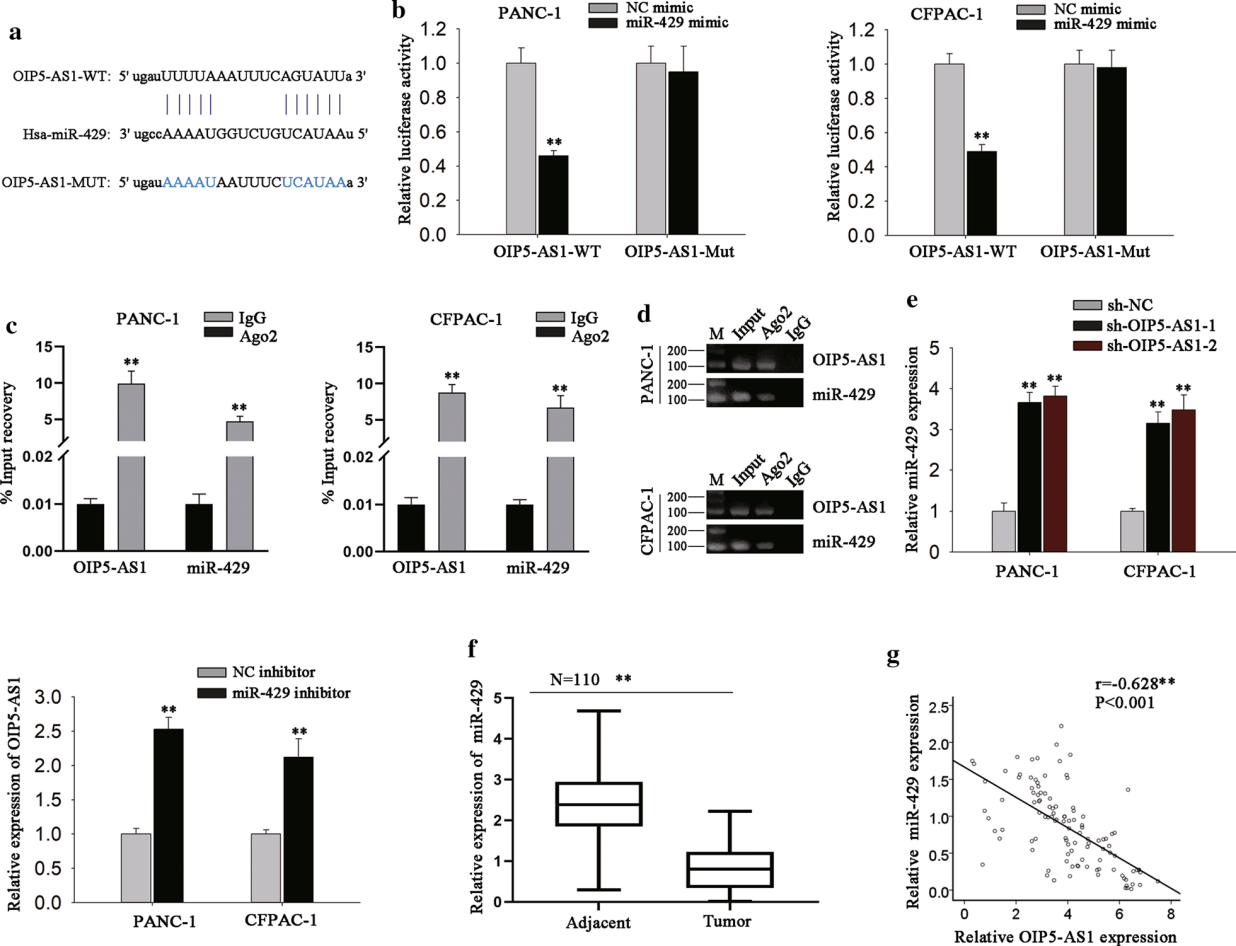
OIP5-AS1 acts as miR-429 sponge and is inversely correlated with miR-429 in PDAC. **a** StarBase conjectured the binding sites between OIP5-AS1 and miR-429. **b** Luciferase activity of indicated reporters under diverse transfections was examined by luciferase reporter assay (**P < 0.01). **c** RIP assay was applied to detect the enrichment of OIP5-AS1 and miR-429 in anti-IgG- or anti-Ago2-induced immunoprecipitates (**P < 0.01). **d** The harvest of above RNAs precipitated in RIP assays were processed with agarose gel electrophoresis after semi-quantitative PCR. **e** RT-qPCR tested the level of OIP5-AS1 and miR-429 in indicated PDAC cells (**P < 0.01). **f** The expression of miR-429 in 110 pairs of PDAC tissues was analyzed by RT-qPCR (**P < 0.01). **g** The correlation between miR-429 and OIP5-AS1 expression in PDAC tissues was tested by Pearson’s correlation analysis (P < 0.001). **P < 0.01 indicated data were statistically significant

Next, we investigated the possible regulatory mechanism of OIP5-AS1 in PDAC. Previous studies have revealed that lncRNAs can serve as ceRNAs by sponging miRNAs [16, 30]. Using bioinformatics database (starBase v3.0), we discovered that there were 62 miRNAs potentially bind with OIP5-AS1 (with the strict stringency and 6 cancer types), of which 11 were significantly upregulated in response to the silencing of OIP5-AS1. Besides, data from starBase 3.0 displayed a negative correlation between OIP5-AS1 and miR-429 in PAAD tissues (Additional file 1: Fig. S1C). Therefore, we chose miR-429 for further analysis. The binding sequence of miR-429 in OIP5-AS1 was shown in Fig. 3a. To further research the interaction between miR-429 and OIP5-AS1, luciferase reporter assay was carried out and results exhibited that miR-429 overexpression considerably inhibited the luciferase activity of the OIP5-AS1-WT, but did not affect that of OIP5-AS1-Mut (Fig. 3b). Recently, lncRNAs have been revealed to function as sponges for certain miRNAs to hinder RISCs-induced mRNA silencing [31]. Therefore, we further performed RIP assay to identify whether the modulation between miR-429 and OIP5-AS1 occurred in RISCs. As a result, we discovered the abundant enrichment of both OIP5-AS1 and miR-429 in Ago2-precipitated pellet compared with IgG control (Fig. 3c), and such result was further validated by the data of agarose gel electrophoresis after semi-quantitative PCR (Fig. 3D). Based on the result of RNA pull-down assay, we confirmed that OIP5-AS1 and miR-429 were enriched in the complexes which were pulled down by miR-429-specific biotin probe (Additional file 1: Fig. S1D). These data confirmed the sponge role of OIP5-AS1 by interacting with miR-429 in PDAC. Additionally, we discovered that miR-429 level was promoted in PDAC cells under OIP5-AS1 depletion, whereas OIP5-AS1 expression was increased by miR-429 inhibitor on the contrary (Fig. 3e). Moreover, the expression of miR-429 expression was lowly expressed in PDAC tissues relative to corresponding non-tumor tissues (Fig. 3f). Furthermore, Pearson’s correlation analysis strongly confirmed the inverse correlation between OIP5-AS1 and miR-429 expression in 110 PDAC tissues (Fig. 3g). In brief, we could conclude that OIP5-AS1 acted as a sponge and negatively modulated miR-429 expression in PDAC.

### MiR-429 targets to FOXD1 in PDAC

**Fig. 4:**
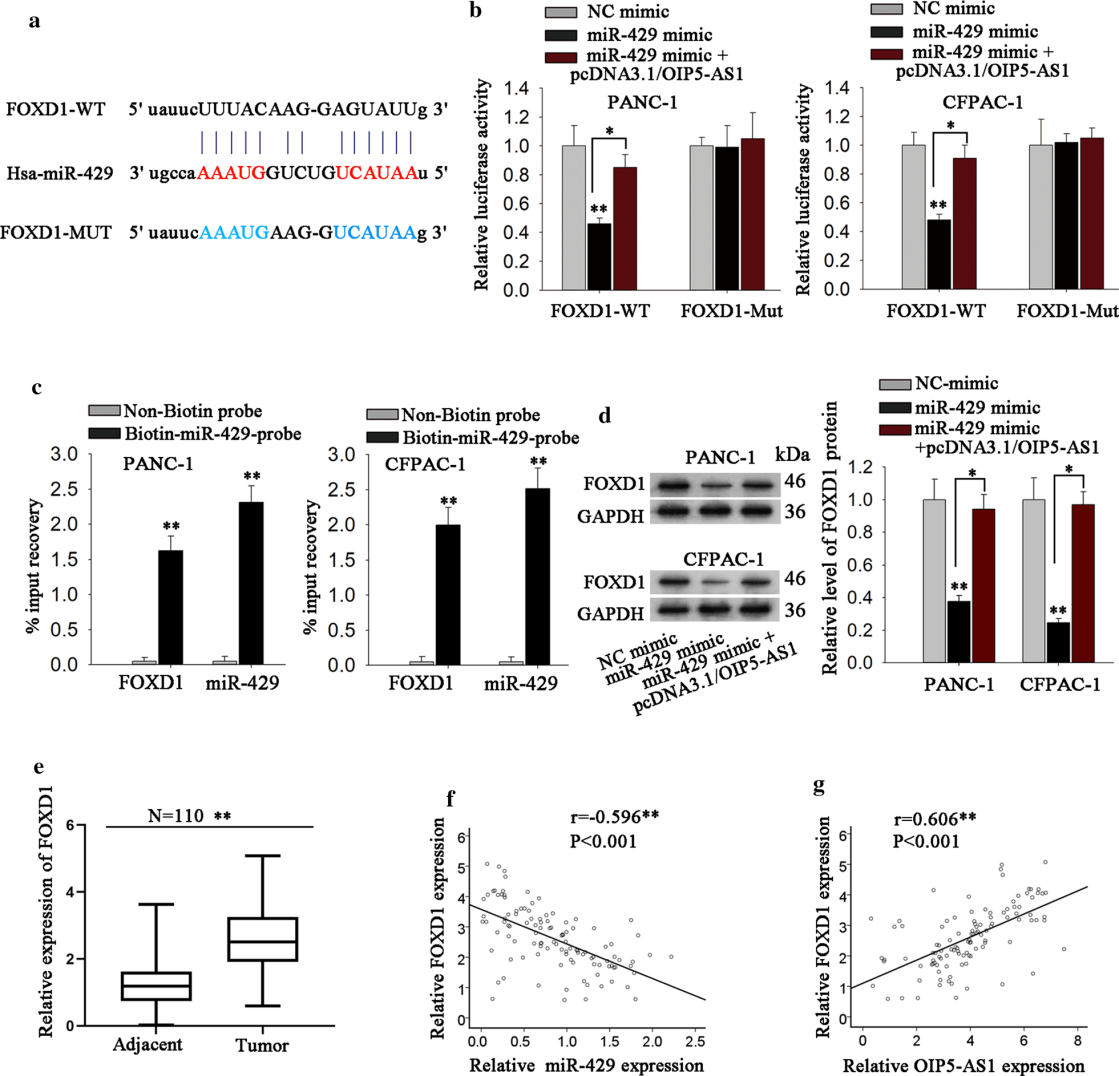
MiR-429 directly targets to FOXD1 and its level is negatively correlated with FOXD1 level. **a** StarBase conjectured the binding sites between FOXD1 3′UTR and miR-429. **b** Luciferase activity in different groups was detected by luciferase reporter assay (*P < 0.05, **P < 0.01). **c** RNA pull-down assay was applied to detect the interaction between FOXD1 and miR-429 (**P < 0.01). **d** The protein expression of FOXD1 in diverse groups was measure by western blot (*P < 0.05, **P < 0.01). **e** RT-qPCR tested FOXD1 expression in 110 pairs of PDAC tissues (**P < 0.01). **f** Pearson’s correlation analysis detected the correlation between miR-429 and FOXD1 level in PDAC tissues (***P < 0.001). **g** Pearson’s correlation analysis unveiled the positive correlation between OIP5-AS1 and FOXD1 expression in PDAC tissues (***P < 0.001). *P< 0.05, **P < 0.01, ***P < 0.001 indicated data were statistically significant

Using bioinformatics tool StarBase v3.0 (10 cancer types, intersection of miRanda and PTTA), the messenger RNAs (mRNAs) that might bind with miR-429 were predicted. Among which, FOXD1 has been identified to have critical influence in human cancers [32, 33]. More importantly, the level of FOXD1 was higher in TCGA PAAD samples (Additional file 2: Fig. S2A), which had a positive correlation with OIP5-AS1 (Additional file 2: Fig. S2B). More importantly, the data of TCGA PAAD samples revealed that high level of FOXD1 indicated poor overall survival of PAAD patients (Additional file 2: Fig. S2C). Thus, FOXD1 was chosen for subsequent experiments. We discovered that FOXD1 had the potential binding sites with miR-429 (Fig. 4a). Besides, it was disclosed that miR-429 up-regulation markedly suppressed the luciferase activity of FOXD1-WT rather than FOXD1-Mut, and this phenomenon could be restored by co-overexpression of OIP5-AS1 (Fig. 4b). Further, RNA pull-down assay verified that both FOXD1 and miR-429 were enriched in the complexes pulled down by biotin-miR-429-probe (Fig. 4c). Subsequently, we detected the specific regulatory role of OIP5-AS1/miR-429 axis in FOXD1 and discovered that miR-429 overexpression resulted in a significant reduction of FOXD1 expression, which could be rescued by OIP5-AS1 up-regulation (Fig. 4d). Moreover, FOXD1 expression was distinctly up-regulated in PDAC tissues (Fig. 4e). Pearson’s correlation analysis exhibited the strong negative correlation between FOXD1 and miR-429 but the close positive association between FOXD1 and OIP5-AS1 level in PDAC tissues (Fig. 4f, g). These evidences indicated that OIP5-AS1, miR-429 and FOXD1 formed a ceRNA network in PDAC.

### OIP5-AS1 positively regulates PDAC progression via regulating miR-429/FOXD1/ERK pathway

**Fig. 5:**
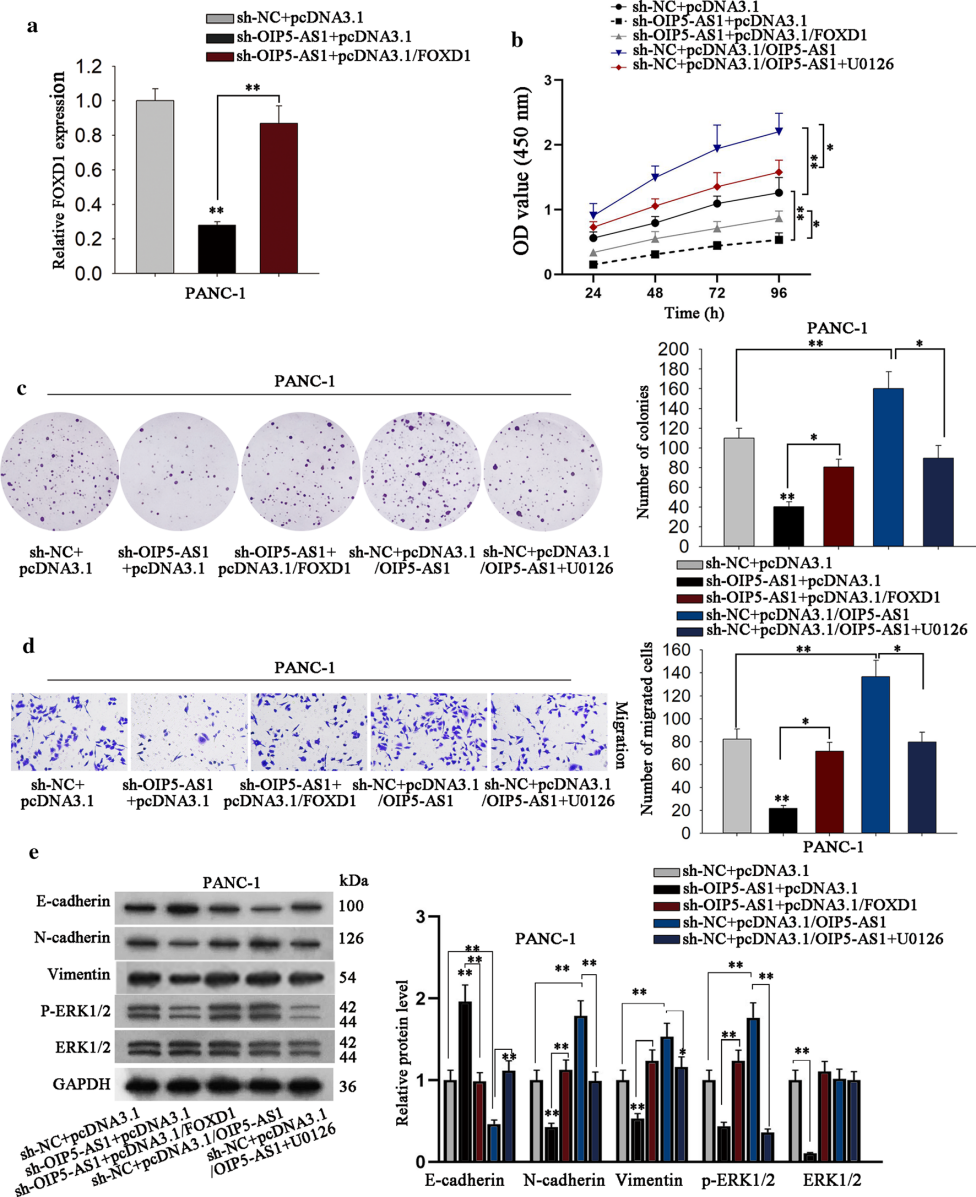
FOXD1 and ERK involve in OIP5-AS1-mediated effects on PDAC cells. **a** RT-qPCR detected the expression of FOXD1 in indicated PANC-1 cells (**P < 0.01). **b**, **c** CCK-8 and colony formation assays detected cell proliferation under different treatments (*P < 0.05, **P < 0.01). **d** Transwell assay detected the cell migration ability under diverse conditions (*P < 0.05, **P < 0.01). **e** EMT process-related markers and ERK pathway-related proteins in corresponding PANC-1 cells were determined by western blot (**P < 0.01). *P < 0.05, **P < 0.01 indicated data were statistically significant

Previously, studies have reported that FOXD1 could activate ERK signaling to accelerate cancer development [34]. Hence, rescue assays were then taken out to explore whether OIP5-AS1 regulated PDAC through modulating FOXD1 and therefore targeting ERK pathway. RT-qPCR detected that overexpression of FOXD1 could recover the reduced level of FOXD1 induced by OIP5-AS1 inhibition (Fig. 5a). Subsequently, CCK-8 and colony formation assays presented that the co-transfection of pcDNA3.1/FOXD1 restored the suppression of sh-OIP5-AS1, whereas the supplement of ERK1/2 inhibitor (U0126) recovered the ascending impacts of pcDNA3.1/OIP5-AS1 on the proliferative ability of PDAC cells (Fig. 5b, c). Besides, the migration of PDAC cells affected by OIP5-AS1 silence or up-regulation was recovered by FOXD1 overexpression or ERK inhibition, respectively (Fig. 5d). Furthermore, the suppression of OIP5-AS1 depletion on EMT process and ERK activation was offset by enhanced FOXD1 expression, and the stimulating effects of OIP5-AS1 up-regulation on EMT process and ERK pathway were counteracted after supplement of U0126 (ERK1/2 inhibitor) (Fig. 5e). Based on these results, we concluded that OIP5-AS1 triggered cell proliferation and migration in PDAC via regulating miR-429/FOXD1/ERK pathway.

### OIP5-AS1 facilitates tumorigenesis of PDAC in vivo

**Fig. 6:**
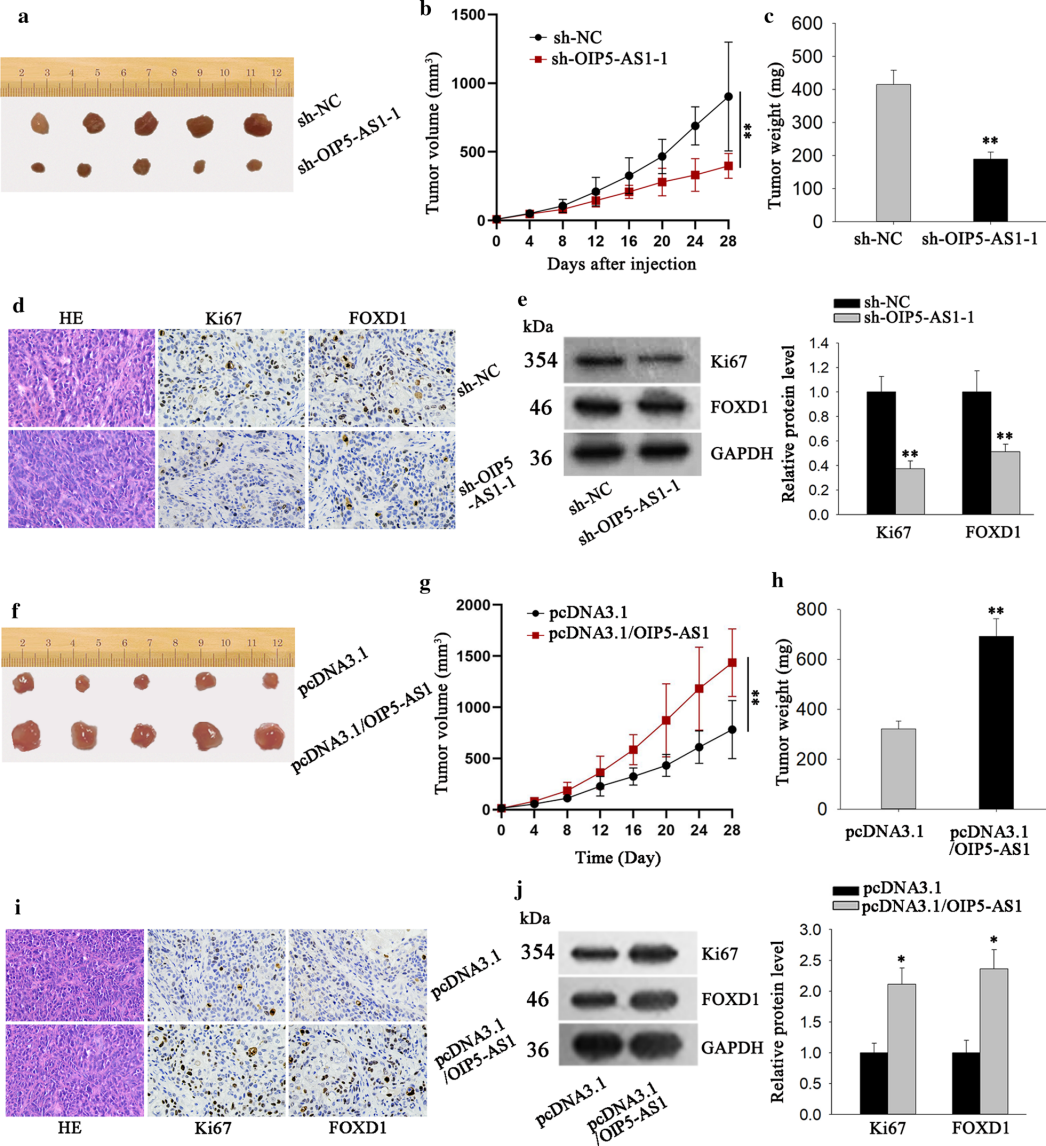
OIP5-ASI facilitates tumor growth in vivo. **a** The representative images of tumors from mice injected with OIP5-AS1-silenced PANC-1 cells or the control cells was shown. **b** The volume of above tumors was detected (**P < 0.01). **c** The weight of above tumors was quantified. **d** Immunohistochemistry was operated to measure Ki67 and FOXD1 expression in tumors from two groups. **e** Western blot assay was adopted to examine the protein level of Ki67 and FOXD1 (**P < 0.01). **f**–**h** Representative images, volume and weight of tumors derived from mice after inoculating CFPAC-1 cells with or without OIP5-AS1 overexpression (**P < 0.01). **i**, **j** The expression of Ki67 and FOXD1 in tumors in Fig. 6f was evaluated by immunohistochemistry assay and western blot (*P < 0.05). *P < 0.05, **P < 0.01 was considered statistically significant

In vivo experiments were implemented to further identify the biological function of OIP5-AS1 in PDAC. Results exhibited that tumors removed from mice injected with OIP5-AS1-silenced PANC-1 cells were conspicuously smaller than those from the mice injected with control cells (Fig. 6a). Moreover, as time went on, the tumors in PANC-1 cells/sh-OIP5-AS1-1 group grown much slower compared with negative control group (Fig. 6b). Additionally, mice injected with PANC-1 cells/sh-OIP5-AS1-1 possessed a lighter tumor weight than those injected with PANC-1 cells/sh-NC (Fig. 6c). Through RT-qPCR analysis, the levels of OIP5-AS1 and miR-429 were found to be separately decreased or increased in sh-OIP5-AS1-1 group (Fig. S3A). IHC results showed that the positivity of the proliferation index (Ki-67) and FOXD1 were reduced in tumors from PANC-1 cells/sh-OIP5-AS1-1 injected mice (Fig. 6d), this result was further demonstrated by western blot analysis (Fig. 6e). On the contrary, tumors originated from mice injected with OIP5-AS1-overexpressed CFPAC-1 cells exhibited bigger sizes with a faster growth rate and heavier weights than those from the control group (Fig. 6f–h). Meanwhile, the we identified the level of OIP5-AS1 was increased in pcDNA3.1/OIP5-AS1 group, while that of miR-429 was significantly reduced (Additional file 3: Fig. S3B). Additionally, the stronger expression of Ki67 as well as higher FOXD1 level were detected in OIP5-AS1-overexpressed group compared to control group (Fig. 6i, j). Taken together, OIP5-AS1 facilitated PDAC cell growth in vivo.

## Discussion

Recently, lncRNAs have been validated to exert vital roles in PDAC. For example, lncRNA THAP9-AS1 promotes PDAC growth via interacting with YAP1 and sponging miR-484 [35]. FEZF1-AS1 facilitates PDAC progression via miR-107/ZNF312B axis [36]. LncRNA OIP5-AS1 is up-regulated in several cancer types and exert oncogenic functions [12–15]. In this work, high level of OIP5-AS1 was determined in TCGA PAAD samples. Based on the database, the prognostic value of OIP5-AS1 was identified in PAAD patients. The expression pattern of OIP5-AS1 and its association with patients’ survival were further validated in 110 collected patient samples. Therefore, we confirmed the research value of OIP5-AS1 in PDAC. Functionally, we also confirmed that OIP5-AS1 involved in PDAC cell growth and migration. All these findings might contribute to unmasking the novel potential therapeutic targets for PDAC (Additional file 4: Table S1).

Increasing evidence indicated that lncRNAs can serve as molecular sponges for miRNAs to modulate the expression of miRNA-targeted genes at post-transcriptional level [37, 38]. LncRNA FLVCR1-AS1 could sponge miR-573 to upregulate E2F3 in lung cancer [39]. LncRNA PVT1 could enhance CDK1 expression via sponging miR-31 in bladder cancer [40]. miR-429 has been reported as a tumor suppressive miRNA reported in several cancers [22–24]. Present study identified hat miR-429 was sequestered and negatively regulated by OIP5-AS1 in PDAC. Numerous researches have emphasized that miRNAs directly targeted to mRNAs, thus regulating the expression of mRNAs. Similarly, miR-429 has been reported as the upstream regulator of mRNAs, thereby functioning as a tumor suppressor [41, 42]. Present study identified FOXD1 as a target of miR-429. FOXD1, located at chromosome 5q12, is a newly discovered member of the FOX transcription factor family [43] and acts as a tumor facilitator in various cancers [33, 44, 45]. Currently, we discovered that FOXD1 interacted with miR-429 and was positively regulated by OIP5-AS1. Besides, FOXD1 expression was negatively associated with miR-429 but positively correlated with OIP5-AS1 expression in PDAC tissues. Intriguingly, there are also evidences indicating the activation of FOXD1 on ERK pathway [32, 46]. Our current study disclosed that OIP5-AS1 upregulated FOXD1, thereby activating ERK pathway in PDAC. Collectively, OIP5-AS1 exerted as a ceRNA to release FOXD1, thus activating ERK pathway in PDAC. Our study unmasked a novel molecular pathway in the progression of PDAC, which might provide new sight into the therapeutic strategy for PDAC.

## Conclusion

OIP5-AS1 facilitated PDAC cell growth both in vitro and in vivo. OIP5-AS1 regulated ERK pathway by targeting miR-429/FOXD1 axis. These findings might help to provide a therapeutic target for PDAC patients (Additional file 5: Table S2).

## Supplementary information

**Additional file 1: Fig. S1.** (A) OIP5-AS1 expression in TCGA PAAD samples and normal samples was obtained from GEPIA (**P < 0.01). (B) Overall survival of PAAD patients with high or low level of OIP5-AS1 in GEPIA database was shown (*P = 0.038). (C) The correlation between OIP5-AS1 and mir-429 expressions in PAAD tissues was obtained from starBase 3.0 (***P < 0.001). (D) RNA pulldown assay detected the enrichment of OIP5-AS1 and miR-429 in biotin-miR-429-probe (**P < 0.01). Non-biotin labeled probe was used as negative control. *P < 0.05, **P < 0.01, ***P < 0.001 indicated data were statistically significant.

**Additional file 2: Fig. S2.** (A) FOXD1 expression in PAAD samples and normal ones was obtained from GEPIA database (**P < 0.01). (B) Correlation analysis of OIP5-AS1 and FOXD1 in TCGA PAAD tissues (*P = 0.016). (C) Overall survival of PAAD patients with high or low level of OIP5-AS1 in GEPIA database was shown (*P = 0.031).

**Additional file 3: Fig. S3.** (A) OIP5-AS1 and miR-429 were detected by RT-qPCR in tumors derived from OIP5-AS1-silenced PANC-1 cells or the control cells (**P < 0.01). (B) The levels of OIP5-AS1 and miR-429 in tumors derived from CFPAC-1 cells with or without OIP5-AS1 overexpression (**P < 0.01).

**Additional file 4: Table S1.** Data of 62 miRNAs potentially bind with OIP5-AS1, from starBase 3.0.

**Additional file 5: Table S2.** Potential targets of miR-429 were predicted by starBase 3.0.

## Data Availability

Research data are not shared.

## Abbreviation

PC: Pancreatic cancer
PDAC: Pancreatic ductal adenocarcinoma
OIP5-AS1: OIP5 antisense RNA 1
lncRNA: Long non-coding RNA
EMT: Epithelial–mesenchymal transition
FOXD1: Forkhead box D1
ERK1/2: Extracellular signal-regulated kinase 1/2
ceRNAs: Competing endogenous RNAs
miRNAs: MicroRNAs
shRNAs: Short hairpin RNAs
RT-qPCR: Quantitative real-time polymerase chain reaction
CCK-8: Cell Counting Kit-8
RIP: RNA immunoprecipitation
RISCs: RNA-induced silencing complexes
IHC: Immunohistochemistry
mRNAs: Messenger RNAs

## References

[1] Kamisawa T, Wood LD, Itoi T, Takaori K (2016). Pancreatic cancer. Lancet.

[2] Siegel R, Naishadham D, Jemal A (2012). Cancer statistics, 2012. CA Cancer J Clin.

[3] Hussain SP (2016). Pancreatic cancer: current progress and future challenges. Int J Biol Sci.

[4] Paulson AS, Tran Cao HS, Tempero MA, Lowy AM (2013). Therapeutic advances in pancreatic cancer. Gastroenterology.

[5] Ponting CP, Oliver PL, Reik W (2009). Evolution and functions of long noncoding RNAs. Cell.

[6] Fatica A, Bozzoni I (2014). Long non-coding RNAs: new players in cell differentiation and development. Nat Rev Genet.

[7] Kondo Y, Shinjo K, Katsushima K (2017). Long non-coding RNAs as an epigenetic regulator in human cancers. Cancer Sci.

[8] Huang F, Chen W, Peng J, Li Y, Zhuang Y, Zhu Z, Shao C, Yang W, Yao H, Zhang S (2018). LncRNA PVT1 triggers Cyto-protective autophagy and promotes pancreatic ductal adenocarcinoma development via the miR-20a-5p/ULK1 Axis. Mol Cancer..

[9] Chen P, Wan D, Zheng D, Zheng Q, Wu F, Zhi Q (2016). Long non-coding RNA UCA1 promotes the tumorigenesis in pancreatic cancer. Biomed Pharmacother.

[10] Li Z, Jiang P, Li J, Peng M, Zhao X, Zhang X, Chen K, Zhang Y, Liu H, Gan L (2018). Tumor-derived exosomal lnc-Sox2ot promotes EMT and stemness by acting as a ceRNA in pancreatic ductal adenocarcinoma. Oncogene.

[11] Ma L, Wang F, Du C, Zhang Z, Guo H, Xie X, Gao H, Zhuang Y, Kornmann M, Gao H (2018). Long non-coding RNA MEG3 functions as a tumour suppressor and has prognostic predictive value in human pancreatic cancer. Oncol Rep.

[12] Yang J, Jiang B, Hai J, Duan S, Dong X, Chen C (2018). Long noncoding RNA opa-interacting protein 5 antisense transcript 1 promotes proliferation and invasion through elevating integrin alpha6 expression by sponging miR-143-3p in cervical cancer. J Cell Biochem.

[13] Wang M, Sun X, Yang Y, Jiao W (2018). Long non-coding RNA OIP5-AS1 promotes proliferation of lung cancer cells and leads to poor prognosis by targeting miR-378a-3p. Thorac Cancer.

[14] Zou Y, Yao S, Chen X, Liu D, Wang J, Yuan X, Rao J, Xiong H, Yu S, Yuan X (2018). LncRNA OIP5-AS1 regulates radioresistance by targeting DYRK1A through miR-369-3p in colorectal cancer cells. Eur J Cell Biol.

[15] Zhang Z, Liu F, Yang F, Liu Y (2018). Kockdown of OIP5-AS1 expression inhibits proliferation, metastasis and EMT progress in hepatoblastoma cells through up-regulating miR-186a-5p and down-regulating ZEB1. Biomed Pharmacother.

[16] Militello G, Weirick T, John D, Doring C, Dimmeler S, Uchida S (2017). Screening and validation of lncRNAs and circRNAs as miRNA sponges. Brief Bioinform.

[17] Ml A, Mp R (2010). MicroRNA: biogenesis, function and role in cancer. Curr Genom.

[18] D’Angelo B, Benedetti E, Cimini A, Giordano A (2016). MicroRNAs: a puzzling tool in cancer diagnostics and therapy. Anticancer Res.

[19] Xu X, Jin S, Ma Y, Fan Z, Yan Z, Li W, Song Q, You W, Lyu Z, Song Y (2017). miR-30a-5p enhances paclitaxel sensitivity in non-small cell lung cancer through targeting BCL-2 expression. J Mol Med (Berl).

[20] Mody HR, Hung SW, Pathak RK, Griffin J, Cruz-Monserrate Z, Govindarajan R (2017). miR-202 diminishes TGFbeta receptors and attenuates TGFbeta1-induced EMT in pancreatic cancer. Mol Cancer Res.

[21] Liu JJ, Zhang X, Wu XH (2018). miR-93 promotes the growth and invasion of prostate cancer by upregulating its target genes TGFBR2, ITGB8, and LATS2. Mol Ther Oncolytics.

[22] Ye ZB, Ma G, Zhao YH, Xiao Y, Zhan Y, Jing C, Gao K, Liu ZH, Yu SJ (2015). miR-429 inhibits migration and invasion of breast cancer cells in vitro. Int J Oncol.

[23] Han Y, Zhao Q, Zhou J, Shi R (2017). miR-429 mediates tumor growth and metastasis in colorectal cancer. Am J Cancer Res.

[24] Song B, Zheng K, Ma H, Liu A, Jing W, Shao C, Li G, Jin G (2015). miR-429 determines poor outcome and inhibits pancreatic ductal adenocarcinoma growth by targeting TBK1. Cell Physiol Biochem.

[25] Iwasaki S, Sasaki HM, Sakaguchi Y, Suzuki T, Tadakuma H, Tomari Y (2015). Defining fundamental steps in the assembly of the Drosophila RNAi enzyme complex. Nature.

[26] Wang H, Liang L, Dong Q, Huan L, He J, Li B, Yang C, Jin H, Wei L, Yu C (2018). Long noncoding RNA miR503HG, a prognostic indicator, inhibits tumor metastasis by regulating the HNRNPA2B1/NF-κB pathway in hepatocellular carcinoma. Theranostics.

[27] Collisson EA, Trejo CL, Silva JM, Gu S, Korkola JE, Heiser LM, Charles R-P, Rabinovich BA, Hann B, Dankort D (2012). A central role for RAF → MEK → ERK signaling in the genesis of pancreatic ductal adenocarcinoma. Cancer Discov.

[28] Bryant KL, Stalnecker CA, Zeitouni D, Klomp JE, Peng S, Tikunov AP, Gunda V, Pierobon M, Waters AM, George SD (2019). Combination of ERK and autophagy inhibition as a treatment approach for pancreatic cancer. Nat Med.

[29] Hanrahan AJ, Solit DB (2012). RAF/MEK dependence of <em> KRAS </em>-mutant pancreatic ductal adenocarcinomas. Cancer Discov.

[30] Beermann J, Piccoli MT, Viereck J, Thum T (2016). Non-coding RNAs in development and disease: background, mechanisms, and therapeutic approaches. Physiol Rev.

[31] Qi X, Zhang D-H, Wu N, Xiao J-H, Wang X, Ma W (2015). ceRNA in cancer: possible functions and clinical implications. J Med Genet.

[32] Pan F, Li M, Chen W (2018). FOXD1 predicts prognosis of colorectal cancer patients and promotes colorectal cancer progression via the ERK 1/2 pathway. Am J Transl Res.

[33] Gao YF, Zhu T, Mao XY, Mao CX, Li L, Yin JY, Zhou HH, Liu ZQ (2017). Silencing of Forkhead box D1 inhibits proliferation and migration in glioma cells. Oncol Rep.

[34] Zhou L, Jia S, Ding G, Zhang M, Yu W, Wu Z, Cao L (2019). Down-regulation of miR-30a-5p is associated with poor prognosis and promotes chemoresistance of gemcitabine in pancreatic ductal adenocarcinoma. J Cancer.

[35] Li N, Yang G, Luo L, Ling L, Wang X, Shi L, Lan J, Jia X, Zhang Q, Long Z (2020). lncRNA THAP9-AS1 promotes pancreatic ductal adenocarcinoma growth and leads to a poor clinical outcome via sponging miR-484 and interacting with YAP. Clin Cancer Res.

[36] Ye H, Zhou Q, Zheng S, Li G, Lin Q, Ye L, Wang Y, Wei L, Zhao X, Li W (2018). FEZF1-AS1/miR-107/ZNF312B axis facilitates progression and Warburg effect in pancreatic ductal adenocarcinoma. Cell Death Dis.

[37] Paraskevopoulou MD, Hatzigeorgiou AG (2016). Analyzing MiRNA-LncRNA interactions. Methods Mol Biol.

[38] Sheng SR, Wu JS, Tang YL, Liang XH (2017). Long noncoding RNAs: emerging regulators of tumor angiogenesis. Future Oncology..

[39] Gao X, Zhao S, Yang X, Zang S, Yuan X (2018). Long non-coding RNA FLVCR1-AS1 contributes to the proliferation and invasion of lung cancer by sponging miR-573 to upregulate the expression of E2F transcription factor 3. Biochem Biophys Res Commun.

[40] Tian Z, Cao S, Li C, Xu M, Wei H, Yang H, Sun Q, Ren Q, Zhang L (2018). LncRNA PVT1 regulates growth, migration, and invasion of bladder cancer by miR-31/CDK1. J Cell Physiol.

[41] Xue H, Tian GY (2018). MiR-429 regulates the metastasis and EMT of HCC cells through targeting RAB23. Arch Biochem Biophys.

[42] Dong H, Hao X, Cui B, Guo M (2017). MiR-429 suppresses glioblastoma multiforme by targeting SOX2. Cell Biochem Funct..

[43] Katoh M, Katoh M (2004). Human FOX gene family. Int J Oncol..

[44] Cheng P, Wang J, Waghmare I, Sartini S, Coviello V, Zhang Z, Kim SH, Mohyeldin A, Pavlyukov MS, Minata M (2016). FOXD1-ALDH1A3 signaling is a determinant for the self-renewal and tumorigenicity of mesenchymal glioma stem cells. Cancer Res.

[45] Li CH, Chang YC, Hsiao M, Liang SM (2019). FOXD1 and Gal-3 form a positive regulatory loop to regulate lung cancer aggressiveness. Cancers.

[46] Ma XL, Shang F, Ni W, Zhu J, Luo B, Zhang YQ (2018). MicroRNA-338-5p plays a tumor suppressor role in glioma through inhibition of the MAPK-signaling pathway by binding to FOXD1. J Cancer Res Clin Oncol.

