# Supplementary figures and images for: LncRNA OIP5-AS1 promotes the malignancy of pancreatic ductal adenocarcinoma via regulating miR-429/FOXD1/ERK pathway
Source: Cancer Cell Int. 2020 Jul 9;20:296. doi: 10.1186/s12935-020-01366-w (PMC7346488; doi:10.1186/s12935-020-01366-w)

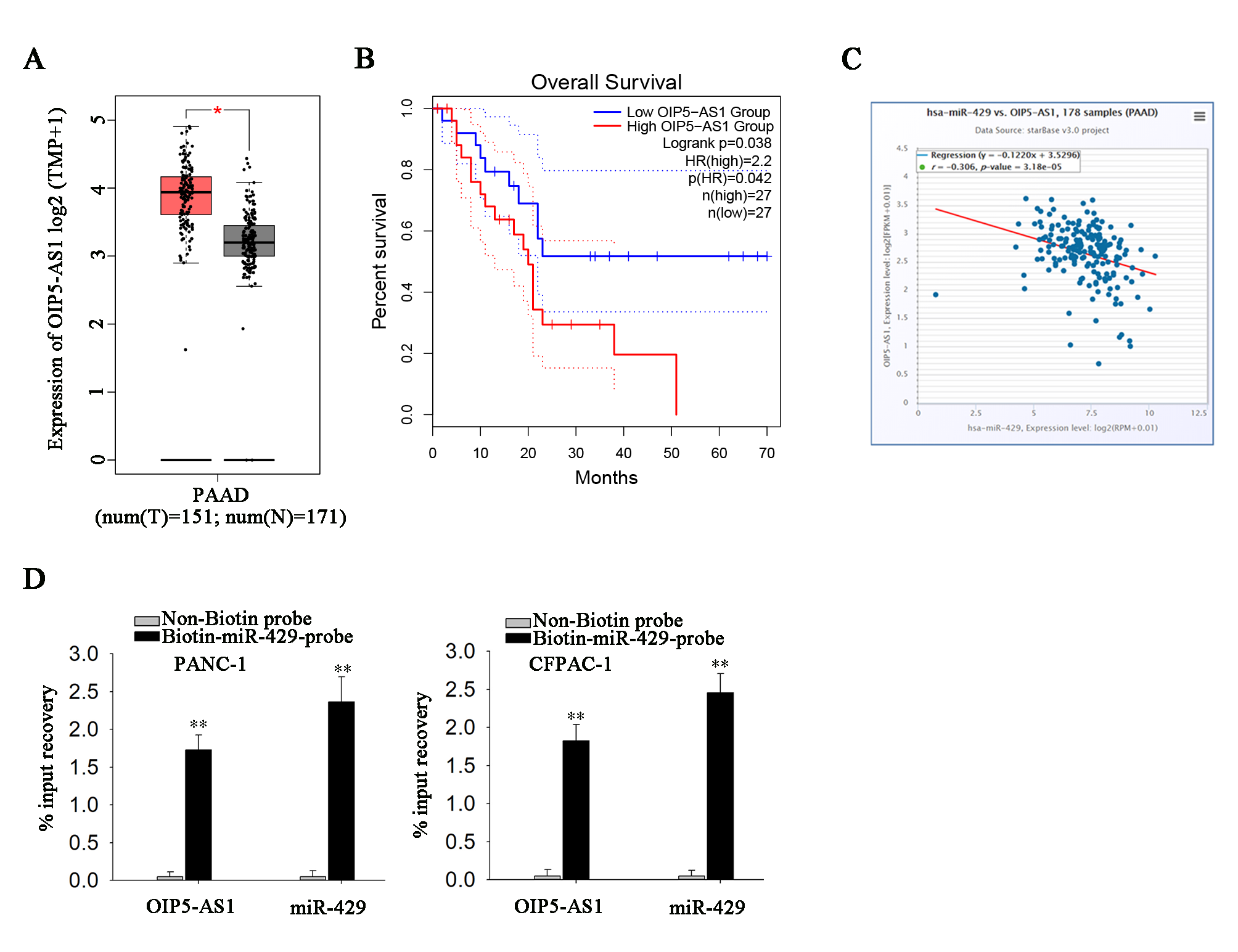

Supplement: Supplementary file 1 — Additional file 1: Fig. S1. (A) OIP5-AS1 expression in TCGA PAAD samples and normal samples was obtained from GEPIA (**P < 0.01). (B) Overall survival of PAAD patients with high or low level of OIP5-AS1 in GEPIA database was shown (*P = 0.038). (C) The correlation between OIP5-AS1 and mir-429 expressions in PAAD tissues was obtained from starBase 3.0 (***P < 0.001). (D) RNA pulldown assay detected the enrichment of OIP5-AS1 and miR-429 in biotin-miR-429-probe (**P < 0.01). Non-biotin labeled probe was used as negative control. *P < 0.05, **P < 0.01, ***P < 0.001 indicated data were statistically significant. [file 12935_2020_1366_MOESM1_ESM.tif]

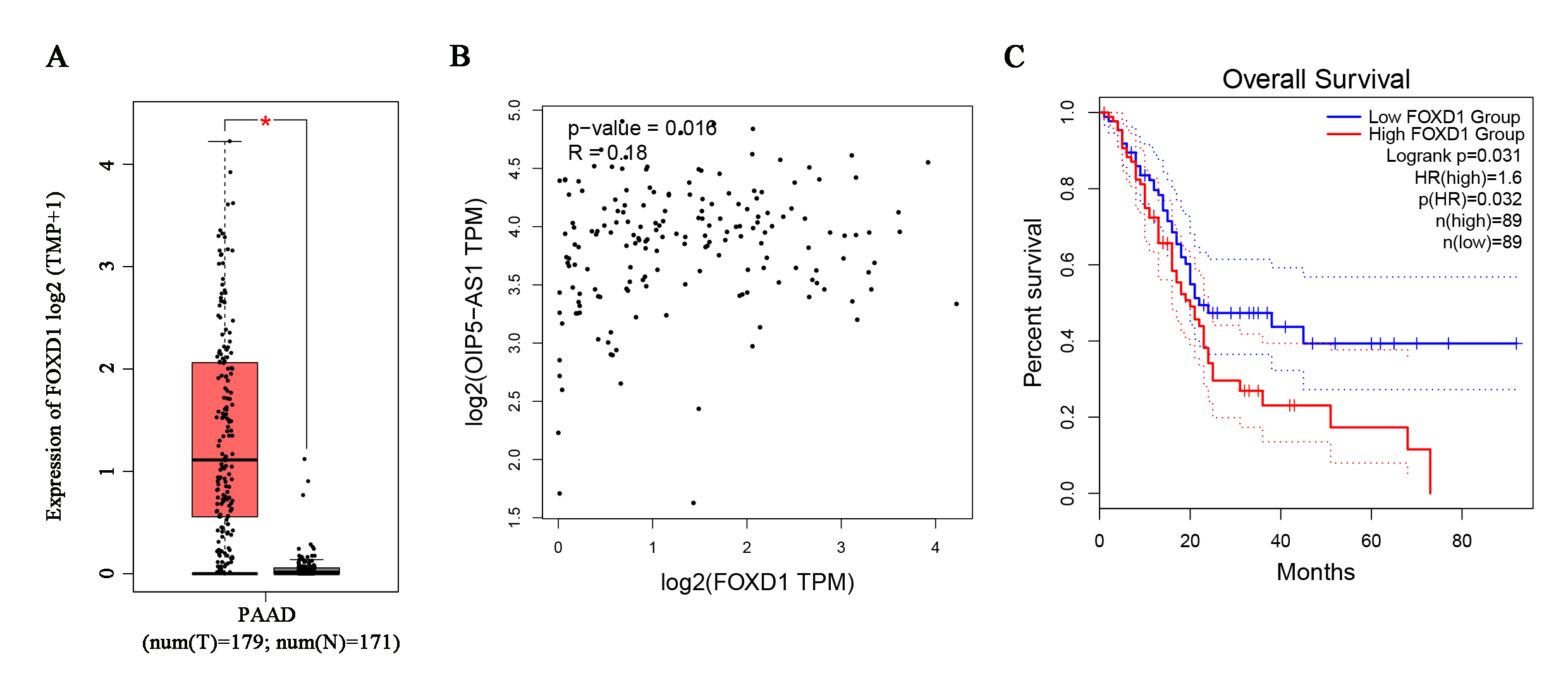

Supplement: Supplementary file 2 — Additional file 2: Fig. S2. (A) FOXD1 expression in PAAD samples and normal ones was obtained from GEPIA database (**P < 0.01). (B) Correlation analysis of OIP5-AS1 and FOXD1 in TCGA PAAD tissues (*P = 0.016). (C) Overall survival of PAAD patients with high or low level of OIP5-AS1 in GEPIA database was shown (*P = 0.031). [file 12935_2020_1366_MOESM2_ESM.tif]

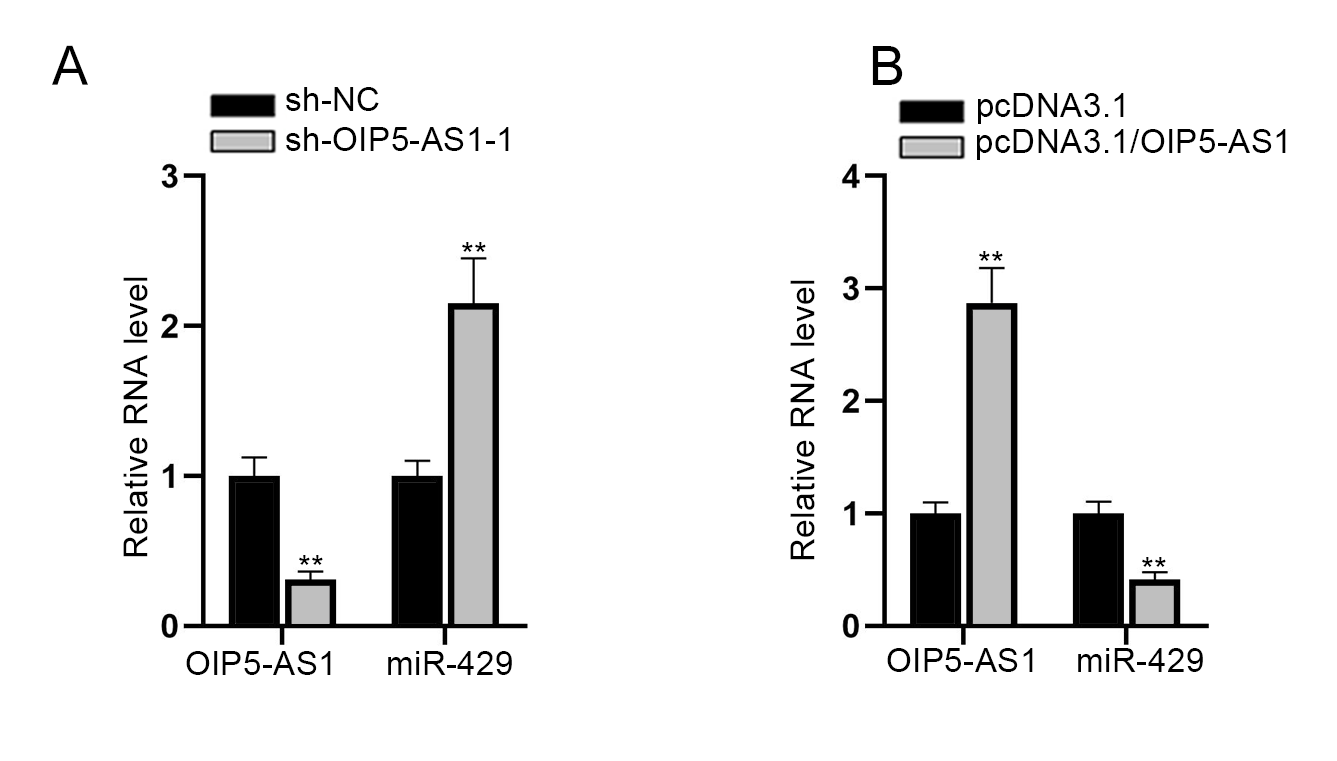

Supplement: Supplementary file 3 — Additional file 3: Fig. S3. (A) OIP5-AS1 and miR-429 were detected by RT-qPCR in tumors derived from OIP5-AS1-silenced PANC-1 cells or the control cells (**P < 0.01). (B) The levels of OIP5-AS1 and miR-429 in tumors derived from CFPAC-1 cells with or without OIP5-AS1 overexpression (**P < 0.01). [file 12935_2020_1366_MOESM3_ESM.tif]
